# Metabolic acidosis in hemodialysis: a neglected problem in Brazil

**DOI:** 10.1590/2175-8239-JBN-2019-0210

**Published:** 2020-04-27

**Authors:** Ana Paula Ramos Silva, Jorge Paulo Strogoff-de-Matos, Jocemir Ronaldo Lugon

**Affiliations:** 1 Universidade Federal Fluminense, Faculdade de Medicina, Departamento de Medicina, Divisão de Nefrologia, Niterói, RJ, Brasil.; 2 Universidade Federal Fluminense, Faculdade de Medicina, Programa de Pós-Graduação em Ciências Médicas, Niterói, RJ, Brasil.

**Keywords:** Renal Dialysis, Renal Replacement Therapy, Acidosis, Diálise Renal, Terapia de Substituição Renal, Acidosis

## Abstract

**Introduction:**

Metabolic acidosis is associated with the high mortality seen in hemodialysis patients. The panorama of metabolic acidosis in hemodialysis in Brazil is unclear since 1996 when the analysis of bicarbonate levels was no longer a compulsory exam. We aimed to establish the prevalence of metabolic acidosis in a hemodialysis population and analyze the factors associated with low bicarbonate levels.

**Methods:**

A cross-sectional study was carried out to assess the prevalence of metabolic acidosis in adults undergoing regular hemodialysis from January to April 2017, in four dialysis centers from Niteroi, Rio de Janeiro, Brazil, and surroundings. For blood gas analysis, samples of 2 mL were collected in heparinized syringes before a midweek dialysis session.

**Results:**

384 patients with a mean age of 58.1 ± 15.8 years (54.5% men and 63.0%, non-white) were included. Approximately 30% had diabetes and 48%, hypertension. Nearly 88% used primary arteriovenous fistula as vascular access. The pre-dialysis mean serum tCO^2^ in the midweek session was 22.7 ± 3.0 mEq/L. The prevalence rate of serum bicarbonate below DOQI recommendation (22 mEq/L or higher) was 40.3%, and 6.5% had serum bicarbonate < 18 mEq/L. The dialyzer use count and the use of low-flux dialyzers were negatively associated whereas age and the standard Kt/V values were positively associated with the serum bicarbonate levels.

**Conclusion:**

The findings were in agreement with global data reported in previous studies. However, because the sample was relatively small and non-representative of the Brazilian population, a more comprehensive study, addressing national data is necessary to substantiate our findings.

## RESUMO

**Introdução:**

A acidose metabólica está associada à elevada mortalidade observada em pacientes em hemodiálise. O panorama da acidose metabólica na hemodiálise no Brasil perdeu visibilidade em 1996, ano em que a análise dos níveis de bicarbonato deixou de ser obrigatória. Nosso objetivo foi estabelecer a prevalência da acidose metabólica em uma população em hemodiálise e analisar os fatores associados a baixos níveis de bicarbonato.

**Métodos:**

O presente estudo transversal avaliou a prevalência de acidose metabólica em adultos regularmente submetidos a hemodiálise de janeiro a abril de 2017, em quatro centros de diálise situados em Niterói e arredores no Estado do Rio de Janeiro. Para análise de gasometria, foram colhidas amostras de 2 mL em seringas heparinizadas antes das sessões de diálise do meio de semana.

**Resultados:**

Foram incluídos 384 pacientes com idade média de 58,1 ± 15,8 anos (54,5% homens; 63,0% não brancos). Aproximadamente 30% tinham diabetes e 48% apresentavam hipertensão. Cerca de 88% usavam fístula arteriovenosa primária como acesso vascular. A concentração sérica média pré-diálise de tCO_2_ na sessão do meio de semana foi de 22,7 ± 3,0 mEq/L. A taxa de prevalência de bicarbonato sérico abaixo do valor recomendado no DOQI (22 mEq/L ou superior) foi de 40,3%; 6,5% dos pacientes apresentaram bicarbonato sérico < 18 mEq/L. Os níveis séricos de bicarbonato apresentaram associações negativas com número de usos do dialisador e uso de dialisadores de baixo fluxo e associações positivas com idade e valores do Kt/V standard.

**Conclusão:**

Os resultados foram concordantes com dados globais relatados em estudos anteriores. Contudo, como a amostra era relativamente pequena e não representativa da população brasileira, são necessários estudos mais abrangentes que venham a abordar dados nacionais para consubstanciar nossos achados.

## INTRODUCTION

The prevalence of end-stage renal disease (ESRD) has been growing worldwide.^1^ Hemodialysis patients have a high mortality rate, with cardiovascular disease accounting for about 50% of the fatalities.^2^ Several studies indicate that the presence of metabolic acidosis is associated with high mortality.^3^^,^ 4 ^,^ 5 ^,^ 6 Conventional hemodialysis treatment may not be sufficient for the adequate control of acidosis in ESRD patients^7^^,^ 8 and complementary interventions, such as oral bicarbonate supplementation, may be required.

Brazil has the third largest number of patients on dialysis of the world with 126,593 people in 2017.^9^ The panorama of metabolic acidosis in hemodialysis in Brazil is unclear, especially because, since 1996, the determination of bicarbonate levels is no longer required as a compulsory laboratory exam in hemodialysis patients, following a Brazilian Ministry of Health ordinance.^10^

The present study aimed to establish the prevalence of metabolic acidosis in a hemodialysis population of four centers in a metropolitan area of Rio de Janeiro, Brazil, and to analyze the factors associated with low bicarbonate levels including demographics, comorbidities, and characteristics of the dialysis treatment.

## METHODS

This was a cross-sectional study of the prevalence of metabolic acidosis in adult patients undergoing regular hemodialysis in Niteroi city and surroundings in the State of Rio de Janeiro. Data were collected from January to April 2017. The convenience sample consisted of patients from four centers who agreed to participate. Patients less than 18 years old, with less than 3 months in hemodialysis, or positive serology for hepatitis and HIV were excluded. The Ethics Committee of the Medical School of Universidade Federal Fluminense approved the study under the number CAAE 57822316900005243 and all patients provided signed informed consent. The study conformed to the principles of the Declaration of Helsinki.

### PROCEDURES

Dialysis centers were numbered from 1 to 4. In centers 1, 2, and 3 dialysis sessions were performed using 4008S hemodialysis machines (Fresenius Medical Care, Schweinfurt, Germany). In center 4, hemodialysis machines consisted of Diamax or Surdial (both made by Nipro Co., Osaka, Japan). Centers 1 and 2 used high-flux polysulfone dialyzers (HF-80S, Fresenius Medical Care, Bad Homburg, Germany); center 3, high-flux polyamix dialyzers (Polyflux 2.1^®^, Gambro, Bad Homburg, Germany); and center 4, low-flux dialyzers (Hemoflow F10 HPS, Fresenius Medical Care, Bad Homburg, Germany). In every center, dialyzers were reprocessed with peracetic acid as the sterilant solution by an automated system for a maximal of 20 times following Brazilian renal replacement therapy regulations. Fresenius Medical Care (Jaguariúna - SP, Brazil) manufactured all the dialysis concentrates used. Characteristics of the dialysis treatment of each center are listed in Table 1.

**Table 1:** Parameters of dialysis treatment

	All	Dialysis centers			
		1	2	3	4
% of patients funded by the PHS	50.5	0	0	68	93
Dialysis hours/week, h	12.3 ± 2.0^a^	13.2 ± 2.4	12.5 ± 2.8	12.1 ± 1.6*	11.8 ± 0.9*
% of patients on > 12h session/week	16.0	36.8	21.4*	7.0* ^Φ^	4.3* ^Φ^
Number of sessions/week	3.3 ± 0.7	3.9 ± 0.9	3.8 ± 0.9*	3.1 ± 0.4* ^Φ^	3.0 ± 0.2* ^Φ^
% of patients on > 4 sessions/week	10.2	21.1	29.8	1.6* ^Φ^	0* ^Φ^
Standard Kt/V	2.22 (1.15-4.21)	2.52 (1.71-3.77)	2.43 (1.15-4.21)	2.19 (1.55-3.55)* ^Φ^	2.13 (1.42-2.94)* ^Φ^
Dialyzer use count at data collection	12.6 ± 5.3	11.1 ± 6.1	12.3 ± 5.2	12.2 ± 5.4	14.0 ± 4.6* ^δ^
Blood flow, mL/min	400 (250 - 500)^b^	400 (250 - 500)	400 (250 - 500)	400 (300 - 450)	350 (250 - 450)
Dialysate flow, mL/min	500	500	500	500	500
Dialysate composition after dilution					
Calcium (2.5/3.0/3.5 mEq/L), %	2.6/97.1/0.3	0/100/0	0/100/0	1.6/98.4/0	7/ 92.8/0.2
Acetate 4.0 mEq/L, %	100	100	100	100	100
HCO_3_- (31.4/32.4 mEq/L), %	(66.7/33.3)	100/0	100/0	0/100	100/0

*PHS:* Public Health System;

a Mean ± S.D.;

b Median(IQR)*;*

* *p <* 0.05 *vs.* Center *1;*

Φ *p <* 0.05 *vs.* Center 2*;*

δ *p <* 0.05 *vs.* Center 3 (differences tested by the chi-square test or ANOVA complemented by the Tukey's test, as appropriate)*.*

For blood gas analysis, samples of 2 mL were collected in heparinized syringes (S-Monovette Blood Collection System, SARSTEDT AG & Co. KG, Nümbrecht, Germany) before a midweek dialysis session. Species were collected from the indwelling needle used to puncture the arterial limb of the arteriovenous fistula or directly from the central intravenous catheter after discarding the volume corresponding to the priming capacity of the device. After collection, the syringe was stored on ice and collectively transported to the laboratory within one hour.

### PARAMETERS AND ESTIMATES

We collected all clinical data and routine laboratory tests from patients’ charts. Blood pressure values were taken as the mean of pre-dialysis measurements of the last three dialysis sessions before enrollment. Routine laboratory tests were extracted from the patient’s chart and calculated as the mean of the last three available values for each patient. Serum albumin was measured by the green bromocresol method. The formula used for calculating standard Kt/V is available online (http://www.hdcn.com/calcf/ley.htm), and requires the urea reduction ratio value for computation.^11^ Values of acid-base parameters were all derived from the blood gas analysis performed as a specific study procedure (ABL5, Radiometer Medical A/S, Denmark).

### STATISTICS

Continuous variables are reported as mean ± S.D. or median and inter-quartile range, as appropriate. Categorical variables are reported as frequencies. The samples were tested for normality with the Kolmogorov-Smirnov test. Differences between centers were tested by the chi-square test and one-way ANOVA or its nonparametric equivalent (Kruskal Wallis ANOVA) complemented by the Tukey’s test. Associations were tested in a logistic regression model using the “Enter” method in which the predictive variables were selected on clinical grounds. *p* values < 0.05 were considered significant. Statistical analyses were performed using SPSS, version 18.0 for Windows (IBM, Chicago, IL, USA).

## RESULTS

The general characteristics of the enrolled patients are shown in Table 2. Three hundred and eighty-four patients were included with a mean age of 58.1 ± 15.8 years, 54.5% men and 63.0% non-white. Mean BMI was 26.0 ± 5.2 kg/m^2^ and 24.6 ± 5.0 kg/m^2^ for men and women, respectively. Thirty percent had diabetes and 48%, hypertension. Nearly 88% used a native arteriovenous fistula as the vascular hemodialysis access. The mean pre-dialysis systolic blood pressure was 147 ± 20 mmHg and the diastolic, 78 ± 13 mmHg. The mean dialysis vintage was 58 ± 55 months; twenty-eight percent were smokers. The laboratory data of the participants are shown in Table 3. The mean total CO_2_ (tCO_2_) was 22.7 ± 3.0 mEq/L, and 40.3% of patients had levels < 22 mEq/L. The prevalence rates of the assigned ranges of tCO_2_ in the whole sample and each dialysis center are shown in Table 4.

**Table 2:** Characteristics of patients (n = 384)

Male gender, n (%)	210 (54.5)
Age, years (mean ± SD)	58.1 ± 15.8
Skin color (White / Non-white), %	37/63
Body mass index, Kg/m^2^ (M/F), (mean ± SD)	26.0 ± 5.2/24.6 ± 5.0
Primary renal disease, n (%)	
*Diabetes mellitus*	116 (30.2)
Hypertension	186 (48.4)
Chronic glomerulopathy	14 (3.6)
Adult polycystic kidney disease	16 (4.2)
Others/indeterminate	52 (13.6)
Vascular access, n (%)	
Native arteriovenous fistula	340 (88.3)
Graft arteriovenous fistula	11 (2.9)
Tunnelled catheter	29 (7.5)
Non-tunnelled catheter	5 (1.3)
SBP / DBP pre-HD, mmHg^a^, (mean ± SD)	147 ± 20/78 ± 13
Dialysis vintage, months, (mean ± SD)	57.9 ± 54.7
Current smoking, n (%)	108 (28.1)

SBP: Systolic blood pressure; DBP: Diastolic blood pressure. aData refer to the means of measurements of each patient before the dialysis session on the day of collection.

**Table 3:** Characteristics of patients - serum laboratory parameters

Albumina, g/dL	4.1 ± 0.4^b^
Calcium^a^, mg/dL	9.2 ± 0.6
Phosphorus^a^, mg/dL	5.1 ± 1.2
Hemoglobin^a^, g/dL	11.3 ± 3.1
iPTH^a^, pg/mL	236 (100 - 526) ^c^
tCO_2_, mEq/L	22.7 ± 3.0

a Data refer to routine measurements calculated as the mean of the last three available results in patients' charts;

b Mean ± S.D.;

c Median (IQR); *iPTH*: intact parathormone.

**Table 4:** Range of pre-dialysis total CO2 (tCO_2_) of the midweek dialysis session in the studied sample

tCO_2_ ranges in mEq/L, n (%)	All	Dialysis centers			
		1	2	3	4
< 18	25 (6.5)	1 (1.8)	1 (1.2)	7 (5.5)	16 (13.9)
18 - < 20	52 (13.5)	3 (5.3)	9 (10.7)	16 (12.5)	24 (20.9)
20 - < 22	78 (20.3)	6 (10.5)	8 (9.5)	34 (26.6)	31 (27.0)
22 - < 24	96 (24.7)	20 (35.1)	17 (20.2)	27 (21.1)	31 (27.0)
24 - < 26	79 (20.6)	16 (28.1)	28 (33.3)	24 (18.8)	11 (9.6)
26 - < 28	43 (11.2)	10 (17.5)	16 (19.0)	15 (11.7)	2 (1.7)
28 - < 30	10 (2.6)	1 (1.8)	4 (4.8)	5 (3.9)	-
≥ 30	1 (0.3)	-	1 (1.2)	-	-
All	384 (100)	57 (100)	84 (100)	128 (100)	115 (100)

A logistic regression model in which the dependent variable was tCO_2_ values < 22 mEq/L is presented in Table 5. Both higher age and higher standard Kt/V were found to be associated with a reduced risk for tCO_2_ values < 22 mEq/L; the dialyzer utilization number in the day of the blood gas analysis and the use of low-flux membrane dialyzers were associated with an increased risk of low tCO_2_.

**Table 5:** Multivariate logistic regression model to test associations of variables with total CO_2_ < 22mEq/L

Predictive variables	OR	95% CI	*p value*
Age (decades)	0.83	0.72-0.98	0.025
Male gender	0.90	0.55-1.48	0.686
*Diabetes mellitus*	0.64	0.35-1.11	0.111
Body mass index (Kg/m^2^)	1.00	0.95-1.05	0.924
Hemoglobin (g/dL)	1.03	0.97-1.11	0.315
Blood flow (mL/min)	0.99	0.95-1.05	0.919
Dialyzer use count	1.19	1.13-1.25	< 0.001
Use of low-flux membrane dialyzers	2.41	1.32-4.40	0.004
Hours of hemodialysis/week	1.08	0.93-1.26	0.277
Standard Kt/V	0.24	0.11-0.55	0.001

O: Odds ratio, C: Confidence interval.

## DISCUSSION

Patients on hemodialysis have a high mortality rate^2^ with several studies indicating a relationship between metabolic acidosis and mortality.^3^^,^ 4 ^,^ 5 ^,^ 6 ^,^ 12 We aimed to determine the prevalence of metabolic acidosis in hemodialysis patients and to analyze the factors associated with low bicarbonate levels in a sample derived from four centers of the metropolitan area of Rio de Janeiro, Brazil.

Fifty percent of the participants of the present study were receiving treatment form the Public Health System, which is markedly lower than the national population under publicly funded treatment (82%).^9^ This aspect may have affected the dialysis prescription: for instance, the fraction of patients on more than 4 sessions of dialysis per week in the present study (10.2%) was strikingly higher than the national average (2.4%).^9^ Perhaps as a reflex of the substantial heterogeneity regarding the source of funding, centers 1 and 2 (exclusively funded by private health insurance companies) had a higher fraction of patients on more than 4 sessions of dialysis per week and higher mean values for standard Kt/V. The most common dialysate calcium concentration was 3.0 mEq/L. Regarding dialysate bicarbonate, only center number 3 used a concentration of 32.4 mEq/L; the remaining centers employed the concentration of 31.4 mEq/L. In the 2018 Brazilian Dialysis Census (data not published yet), the national median of the bicarbonate concentration of the dialysate was 32 mEq/L, a number very close to the ones used in the studied centers. In a comprehensive multinational study, dialysate bicarbonate levels were grouped as values ≤ 32 mEq/L, 33-37 mEq/L, and ≥ 38 mEq/L, used at a frequency of 15%, 60%, and 25%, respectively.^4^ According to such classification, data of the present study (as the vast majority of Brazilian centers) seats in the low range of the dialysate bicarbonate concentration used worldwide.

The present study included 384 patients, with a demographic profile comparable to the ones reported in the last Brazilian Dialysis Census.^9^ Judging from the mean values of Kt/V and body mass index, and from the mean serum levels of albumin, calcium, phosphorus, iPTH, and hemoglobin, participants received a dialysis treatment well within the standards of adequacy on average.^13^ The mean pre-dialysis serum tCO_2_ in a midweek session of our sample (22.7 mEq/L) was very similar to global data reported in 2 studies, 21.9 mEq/L^3^ and 22.9 mEq/L.^4^

As a whole, 40.3% of patients had serum bicarbonate levels below DOQI recommendation (22 mEq/L).^14^ Values >27 mEq/L and < 18mEq/L, which had already been associated with higher mortality and higher hospitalization rate,^3^^,^ 8 were found in 14.1% and 6.5% respectively. Once more, results varied between centers, with the highest mean value for tCO_2_ in center 1, and the lowest one in center 4. Discrepancies in the dialysis prescription may again have accounted for these differences: for instance, the center with the lower mean tCO_2_ values was the only one in which all patients underwent three dialysis sessions per week.

A logistic regression model was designed to test associations with tCO_2_ below 22 mEq/L, which included the following variables: age in decades, gender, diabetes, body mass index, hemoglobin, blood flow, dialyzer use count, use of low-flux membrane dialyzers, hours of dialysis per week, and standard Kt/V. Four variables in the model showed a statistically significant association with a blood tCO_2_ below 22 mEq/L: older age and higher standard Kt/V were protective factors whereas higher dialyzer use count and the use of low-flux membrane dialyzers were associated with increased risk.

The negative impact of dialyzer reuse and the use of low flux membrane dialyzer on serum bicarbonate, even after extensive adjustment, did come as a surprise and should await further confirmation. The dialyzer reuse does not seem to affect the effectiveness of dialysis and is a widely used practice in countries with restricted resources.^15^^,^ 16 In addition, although some studies show that the all-cause mortality in patients undergoing high flux dialysis may be lower compared to low flux hemodialysis,^15^^,^ 17 this subject is still a matter of controversy.^18^

The association of age and higher levels of bicarbonate had already been reported^3^^,^ 18 and can be interpreted as a consequence of the lower acid generation in the dialysis interval due to the sarcopenia and reduced physical activity that can affect older hemodialysis patients. Although higher values for Kt/V 3 ^,^ 19 and duration of dialysis session^18^ have been individually associated with higher levels of serum bicarbonate, we could not find studies addressing the role of the standard Kt/V in this regard. However, higher values of standard Kt/V can conceivably be associated with higher serum bicarbonate values taking in consideration that its calculation requires the last two mentioned variables (the Kt/V itself and the weekly frequency of dialysis).

The study presents some limitations, the main one being a relatively small sample not representative of the Brazilian population. These limitations restrict the generalizability of the findings; however, the information generated regarding this important and unexplored subject in Brazil, the metabolic acidosis in maintenance hemodialysis, is of great value.

## CONCLUSIONS

The mean pre-dialysis serum tCO_2_ in a midweek session of the studied sample was 22.7 mEq/L. The prevalence rate of serum bicarbonate below DOQI recommendation (22mEq/L or higher) was 40.3%, and 6.5% had serum bicarbonate below 18 mEq/L. The dialyzer use count and the use of low-flux membrane dialyzers were negatively associated with serum bicarbonate levels. Older age and higher standard Kt/V values were positively associated with serum bicarbonate levels.

Our findings conform to global data reported in previous studies but a more comprehensive study addressing national data is necessary to substantiate our findings.
